# The Association of Nonsuicidal Self-Injury with Quality of Life and Mental Disorders in Clinical Adolescents—A Network Approach

**DOI:** 10.3390/ijerph18041840

**Published:** 2021-02-14

**Authors:** Dora Gyori, Bernadett Frida Farkas, Lili Olga Horvath, Daniel Komaromy, Gergely Meszaros, Dora Szentivanyi, Judit Balazs

**Affiliations:** 1 Doctoral School of Psychology, Eotvos Lorand University, 1075 Budapest, Hungary; horvath.lili@ppk.elte.hu (L.O.H.); szentivanyi.dora@ppk.elte.hu (D.S.); 2 Institute of Psychology, Eotvos Lorand University, 1075 Budapest, Hungary; komadani@gmail.com (D.K.); balazs.judit@ppk.elte.hu (J.B.); 3 Mental Health Sciences Doctoral School, Semmelweis University, 1083 Budapest, Hungary; missfarkasdetti@gmail.com (B.F.F.); meszaros.gergely.83@gmail.com (G.M.); 4 Faculty of Social and Behavioural Sciences, University of Amsterdam, 1018 WV Amsterdam, The Netherlands; 5 Department of Behavioural and Movement Sciences, Vrije Universiteit, 1081 HV Amsterdam, The Netherlands; 6 Faculty of Medicine, Department of Psychiatry and Psychotherapy, Semmelweis University, 1083 Budapest, Hungary; 7 Pedagogical Assistance Services, 1067 Budapest, Hungary; 8 Department of Psychology, Bjørknes University College, 0456 Oslo, Norway

**Keywords:** non-suicidal self-injury, quality of life, mental disorders, adolescents, self-report, parent-report, network approach

## Abstract

Although earlier research has highlighted that psychiatric disorders significantly impair patients’ quality of life (QoL), few studies have examined the relationship between nonsuicidal self-injury (NSSI) and QoL. Our aim was to investigate whether QoL mediates the mental disorder–NSSI relationship, and to study the QoL ratings agreement of self and parents in a clinical population of adolescents. We involved 202 adolescents from Vadaskert Child Psychiatric Hospital and Outpatient Clinic, Budapest, aged 13–18 years. All participants completed the Deliberate Self-Harm Inventory, Inventar zur Erfassung der Lebensqualität bei Kindern und Jugendlichen, and the Mini International Neuropsychiatric Interview Kid. To map the interrelationship between the NSSI, mental disorders, and QoL dimensions, Mixed Graphical Models were estimated. Adolescents with a history of NSSI rated their QoL to be significantly lower than adolescents without NSSI. Self and parents’ QoL ratings are closer in the NSSI sample than in the no-NSSI sample. Among all QoL dimensions, only family problems had a direct significant association with NSSI engagement. Our results highlight that, contrary to our hypothesis, the presence of mental disorders mediates the relationship between most QoL dimensions and the occurrence of NSSI. Our results draw attention to the potential causal effect of environmental factors (e.g., peer problems) on mental disorders that, in turn, result in NSSI. The present paper highlights the importance of network modelling in clinical research.

## 1. Introduction

NSSI refers to the intentional destruction of body tissue without suicidal intent (such as cutting, burning, scraping skin, hitting, and biting oneself) and for purposes not culturally sanctioned [1]. Nonsuicidal self-injury disorder (NSSI-D) has been proposed as an individual new diagnostic entity in the Diagnostic and Statistical Manual of Mental Disorders 5th Edition (DSM-5), under section III, ”Conditions for Further Study” [2]. The typical age onset of NSSI is 12–14 years [3]; its prevalence increases in young adolescence and decreases in late adolescence [4]. Lifetime prevalence of NSSI in the community adolescent population has been found to be 17–46.5% [5,6,7,8], although in adolescent psychiatric samples the rate is much higher at 60–80% [9,10]. Despite a decline in late adolescence, repetitive NSSI during the adolescent years indicates a high risk of long-term dysfunctional emotion regulation strategies, suicidality, and other risk-taking behaviours (e.g., substance misuse) [11]. According to most studies, NSSI is more commonly seen among women, and this gender difference is larger in clinical samples compared to the general population [12]. It has been suggested that NSSI is associated with several internalising and externalising disorders [3,13,14], as well as being a significant risk factor for suicidal behaviour [15,16,17]. The comorbidity of suicidal behaviour in community samples is approximately 50% [18], but in clinical samples it is approximately 70% [19]. Due to these findings, NSSI is an important public health concern for young people [20].

The quality of life (QoL) measurement in psychiatric populations provides relevant and additional information about a patient’s functional adaptation [21,22]. A well-respected definition of QoL is: “individuals’ perception of their position in life in the context of the culture and value systems in which they live and in relation to their goals, expectations, standards and concerns” [23] (p. 1403). People’s satisfaction with their everyday life is subjective [21,23]. Moreover, mental health difficulties influence a person’s ability to deal with daily activities, and this can affect their general sense of well-being [24]; however, QoL is a broader concept than functional impairment related to psychiatric disorders [25]. It is a multidimensional concept [23] and includes the individual’s subjective perception of well-being across all domains of life, including the physical, psychological, social, and emotional contexts [26]. In QoL measurements, besides the patients themselves, relevant others such as parents can also provide information [21,26,27]. However, it has been proven that parent–child ratings related to the child’s symptoms may not agree on many issues [21,24,25,26]. Furthermore, parents tend to more highly rate the negative effect of psychiatric disorders and evaluate a lower QoL for their children compared to the child’s rating [21,24,26].

Over the last decade, there has been growing evidence that children with psychiatric disorders, such as anxiety and mood disorders [21,25,26], and externalising disorders, such as oppositional defiant disorders (ODD), conduct disorder (CD) [28], and attention-deficit hyperactivity disorder (ADHD), reduce the QoL of children and young people [24,25,28,29,30]. Furthermore, in a clinical adolescent sample, Balazs et al. found that QoL significantly mediates the relationship between internalising psychopathology and peer problems, as well as suicidal risk [31]. According to these results, there has been growing interest in the QoL of children and adolescents with psychiatric disorders [26,27], but until now very few studies have focused on the relationship between NSSI and QoL in an adolescent sample.

We primarily focused on studies that measured self-injurious behaviour without suicidal intent, but because of the great conceptual heterogeneity of self-injury definition and very few studies related to this topic, we included studies with both NSSI and deliberate self-harm definitions.

Rönkä et al. (2013) explored the associations of deliberate self-harm (DSH) with loneliness, self-rated health, and life satisfaction in adolescence (*n* = 7014, *m* (mean age) = 15.5 years) from the Northern Finland Birth Cohort 1986 [32]. Their study was based on the following definition: DSH is an act with a non-fatal outcome in which an individual deliberately initiates a behaviour (such as self-cutting or jumping from height), ingests a substance (medicines/drugs), or ingests a non-ingestible substance or object with intent to harm the self [33]. Satisfaction with life was measured with a single question: “What is your opinion about your current life situation?”, and self-rated health was assessed with the question “How would you describe your health?”. According to their results, DSH was associated with loneliness and dissatisfaction with life in both genders [32]. Similarly, Zullig (2016) explored the association among DSH, seven life satisfaction domains (family, friends, school, self, living environment, romantic relationships, and physical appearance) and health-related quality of life (HRQoL) among college students (*n* = 723, *m* = 20 years, SD (standard deviation) = 1.01). DSH was measured using a single question based on the definition of DSH (intentional direct injuring of body tissue without suicidal intent [19]; their results revealed a negative association between DSH and overall life satisfaction. Students who engaged in DSH were mostly dissatisfied with their friendships and themselves [34]. Kaess et al. (2017) measured the relationship between HRQoL and psychopathology in adolescents (*n* = 264, 12–17 years) with both subthreshold and full-syndrome Borderline Personality Disorder (BPD) and without BPD. Adolescents with subthreshold and full-syndrome BPD reported lower HRQoL compared to those without BPD; results showed that the frequency of self-injury has a negative influence on HRQoL [35].

We would like to highlight the importance of network modelling in clinical research. We plan to test a hypothesised explanatory mechanism, namely whether the different QoL dimensions mediated the association between mental disorders and NSSI. The inclusion of all QoL domains and mental disorders in one model is necessary for several reasons. First, estimating different models for each mental disorder and applying *p*-value corrections would lead to a reduction in statistical power [36]. Second, comorbidity is often tremendously high between mental disorders [37], including suicidality [38]. Hence, separate models for each disorder would yield strongly biased parameter estimates due to problems associated with the omitted variables (the presence of certain disorders is strongly linked to the presence of others). Nevertheless, even including all mental disorders as independent variables (IV) and all QoL dimensions as mediators could lead to incorrect model specifications. Theoretically, distinct QoL dimensions, as well as different mental disorders and self-harming behaviours, could influence each other in different pathways. For instance, it is possible that attention-disruptive disorders lead to school problems that, in turn, cause family problems that, finally, are linked to NSSI. A classical mediator model would not be able to test these sort of relationships, only the predefined independent-mediator-dependent variable (DV) paths. In sum, classical mediation analysis would lack statistical power and would yield highly biased results due to omitted variables and model misspecification. On the contrary, psychological networks provide a comprehensive framework to model these sorts of complex interrelationships.

Networks are abstract models comprising a set of nodes and edges; the former provide information about the relationship between the latter [39]. In psychological networks, nodes represent, for example, mood states or symptoms, while edge weights speak for the statistical relationships between them [40]. If the variables (nodes) follow a multivariate normal distribution (this assumption will be relaxed later), these relationships are often partial correlation coefficients [41,42]. Partial correlation coefficients represent conditional independence associations: in these networks, edge weights represent a correlation between two variables over and beyond the effect of all others. The lack of an edge between two nodes means that the two variables are conditionally independent (there is no association between them after controlling for all other variables).

Partial correlation networks, as a result, are better tools to estimate predictive mediation than multiple regression analysis [43]; they detect the (direct and indirect) predictors of *all* variables instead of prespecifying roles as IVs, mediators, and DV. Thus, in network models, more complex pathways can be revealed than in mediator models. Furthermore, considering both a mediation model estimated on observational (non-experimental) cross-sectional data, where variable X is the IV and variable Z is the mediator, and another in which the roles are reversed, it is highly likely that both models will result in significant mediating effects. On the contrary, a network where variables X and Y are not directly related, but are indirectly connected by Z (X-Z-Y), shows that X and Y are correlated, but any predictive effect between the two is mediated by Z. This sort of conditional independence plays a crucial role in causal inference. When all relevant variables are observed, a non-zero edge between X and Z means that X causes Z, Z causes X, there is a reciprocal link between the two nodes, or both of them cause a third variable in the network [44,45].

Nevertheless, due to sampling variation, network edges between nodes will never be exactly zero. Some correlations will be spurious, in other words, false positives [46]; these are due to chance patterns in the sample and will have to be removed. If one wants to avoid the loss of statistical power [46], an alternative to multiple testing/correction is the “least absolute shrinkage and selection operator” (LASSO) [47]. This is a regularisation technique borrowed from the field of machine learning.

In the present research we use network modelling as methodological framework.

The aim of the current study was to explore the complex relationship between NSSI, mental disorders, and different QoL dimensions in a clinical sample of adolescents using complex modelling techniques.

Our hypotheses were as follows:

**Hypothesis** **1.** *Adolescents who engaged in NSSI will report lower QoL than adolescents without NSSI*.

**Hypothesis** **2.** *Parents of adolescents who engaged in NSSI will rate their children’s QoL level to be lower than their adolescents’ self-rating level of QoL.*

**Hypothesis** **3.** *The association between mental disorders and NSSI is mediated by QoL.*

## 2. Materials and Methods

Detailed study information related to methodology (such as procedure, ethics, and subjects) has already been published and described previously [48,49,50]. In the subsequent chapters, we detail relevant information related to methods.

### 2.1. Ethics

The study was conducted in accordance with the Declaration of Helsinki, and the protocol was approved by the Ethical Committee of the Medical Research Council, Hungary (ETT-TUKEB) (protocol number: 5750/2015/EKU). After the nature of the study had been explained both to the adolescents and their parents, all participants gave their written informed consent for inclusion before they participated in the study. A code–decode system was used to identify those adolescents who had suicidal risk according to the structured psychiatric diagnostic interview (as described in due course). In this case, parents and adolescents were informed, and participants were referred to the healthcare system.

### 2.2. Subjects

A clinical adolescent sample was involved in the study. The clinical group was enrolled in the Vadaskert Child and Adolescent Psychiatric Hospital and Outpatient Clinic, Budapest, Hungary, during a recruitment period spanning 25 February, 2015 to 9 May, 2016. According to the inclusion criteria, adolescents were aged between 13–18 years and had to be psychiatric inpatients in the acute inpatient ward. Exclusion criteria were the following: lack of sufficient Hungarian language skills, serious psychiatric states, and mental retardation preventing the completion of self-administered questionnaires.

### 2.3. Measures

Psychiatric diagnoses were measured with the modified version of the Hungarian Mini International Neuropsychiatric Interview Kid (MINI Kid) [51,52,53,54]. The MINI Kid is a comprehensive structured diagnostic interview which assesses the major child and adolescent psychiatric disorders: mood disorders (i.e., major depressive episodes, dysthymic disorder, hypo/manic episodes), anxiety disorders (i.e., panic disorders, agoraphobia, separation anxiety disorder, social anxiety disorder, specific phobia, post-traumatic stress disorder (PTSD), generalised anxiety disorder (GAD)), obsessive-compulsive disorder (OCD), alcohol abuse/dependence and psychoactive substance abuse/dependence, tics, Tourette’s disorder, ADHD, CD, ODD, anorexia nervosa, bulimia nervosa, psychotic disorders, suicidality and adjustment disorder. Before the study, interviewers received training, and during the investigation they were regularly supervised.

Balazs and colleagues (2004) validated the Hungarian version of the MINI Kid [54]. We applied this version in our study. According to the psychometric properties of the Hungarian version, both interrater and test-retest reliability was adequate. Cohen’s kappa coefficient (κ) of interrater reliability in the case of most psychiatric disorders was very good (κ > 0.80), in the case of one disorder was good (κ > 0.73). Test-retest reliability in case of 52% of psychiatric diagnoses was excellent (κ > 0.80), in 40% of psychiatric disorders was very good (κ > 0.60), in one case the value was moderate (κ = 0.46), in another case was poor (κ > 0.36). The result of criterion validity was also reported acceptable. In 61.5% of examined disorders, the sensitivity was found very good or good. According to validity analysis, poor sensitivity was not found. In the case of 89.5% of psychiatric diagnoses, the specificity was also found very good.

The Deliberate Self-Harm Inventory (DSHI) [55] was used to measure NSSI. The DSHI is based on the conceptual definition of NSSI as deliberate, direct destruction of body tissue without suicidal intent [56]. The DSHI is a self-rated questionnaire which assesses different types of self-harming behaviour, with 17 items (such as cutting, burning, carving, scratching, biting, and other forms of self-harm) being answered by a “yes” or “no”. We used the shortened version of DSHI questionnaire [57] first within the EU FP7 funded project, Saving and Empowering Young Lives in Europe (SEYLE). The high quality of the Hungarian translation of the 17-item DSHI [55] was ensured with a multistep translation procedure, including initial translation from English to Hungarian and back-translation from Hungarian to English, harmonisation, linguistically adaptation and pilot study. The final Hungarian version of the questionnaire, which was applied in this study was already used in other several studies of our research group [48,49,50].

QoL was examined by the Hungarian adolescent self-reported version of the Inventar zur Erfassung der Lebensqualität bei Kindern und Jugendlichen (ILK) self-report questionnaire [58,59]. The questionnaire includes one item for global well-being and six different domains in regard to school, family, peer relations, alone activities, physical health, and mental health. Both adolescent and parent self-report versions use a 5-point Likert-type scale (with 1 being the best and 5 the worst QoL). The ILK can be used for both healthy and ill populations.

Kiss and colleagues (2007) reported the psychometric properties of the Hungarian version of ILK [58]. Both the reliability and validity of Hungarian version of ILK questionnaire were found adequate during the adaptation process. The internal consistency reliability was found for adolescents’ version Cronbach: α = 0.73 and for parents’ version Cronbach: α = 0.78. Interrater reliability of adolescents-parents rating was Pearson correlation (r) r = (between) 0.28–0.62, test-retest reliability was reported with intraclass correlation coefficient (ICC), ICC = (between) 0.54–0.78 for the parents’ version, ICC = (between) 0.57–0.71 for adolescents’ version. Discriminant validity was found also adequate.

### 2.4. Statistical Analysis

Statistical analysis was performed using R (3.5.1 version, R Foundation for Statistical Computing, Vienna, Austria). Because of the low prevalence data of psychiatric diagnoses, the following diagnoses were excluded: Tourette’s syndrome, tics, anorexia nervosa, bulimia nervosa, and autism spectrum disorder. The following grouped diagnoses were involved in our analysis: (1) mood disorders: major depressive episodes, dysthymic disorder, and hypo/manic episode; (2) anxiety disorders: panic disorder, agoraphobia, separation anxiety disorder, social anxiety disorder, specific phobias, PTSD, GAD, and OCD; (3) attention-disruptive disorders: ADHD, CD, and ODD; (4) substance use disorders: alcohol as well as psychoactive substance abuse and dependence; (5) psychotic disorders; and (6) suicidality. In our analysis, the NSSI variable was assessed with the presence or absence of any DSHI item. QoL variables consist of global well-being and the six different domains (school, family, peer relations, alone activities, physical health, and mental health) from the ILK questionnaire.

In testing the first two hypotheses, an α-level below 0.5 was considered to be significant. Descriptive statistics are reported. To test hypothesis 1 (to compare the mean of each QoL dimension in the NSSI and the no-NSSI groups), two-sample independent t-tests were used. To test hypothesis 2 (compare the self and parent ratings in each QoL dimension groups), paired sample t-tests were conducted for all seven QoL domains. To account for the potential bias arising from multiple testing, a Bonferroni correction was applied for the *p*-values. Both original and corrected *p*-values are reported in this section.

In Hypothesis 3, we use network modelling in order to test a hypothesised explanatory mechanism, namely whether the different QoL dimensions mediated the association between mental disorders and NSSI.

Regularised networks have been widely used in clinical psychology [60,61,62,63,64,65] and psychiatry [42,66,67]. LASSO regularisation maximises the sum of absolute correlations with the help of a lambda parameter [68]. Consequently, compared to an unregularised network, all parameter estimates decrease; small ones become exactly zero and so the network becomes sparser. Too low values of lambda lead to the retention of false positive edges and too high lambdas result in the removal of true edges. Hence, the goal is to minimise the number of false positive edges and maximise the number of true ones [69,70]. To implement this, multiple network models are estimated with differing lambda values [71] and model selection is carried out based on certain criterion. One of them, the Expected Bayesian Information Criterion (EBIC) [72], has been shown to perform especially well in retrieving the true network structure [69,70,73]. In estimations with EBIC, the gamma hyperparameter, usually set between 0 and 0.5 [70], determines the simplicity of the models; lower values lead to more parsimonious networks. It is important to note that the LASSO technique maximises specificity, hence instead of aiming to reduce the number of false negatives, it seeks to include as few false positive edges as possible. In other words, a missing edge is not irrefutable proof of the lack of a true relationship [43]. This phenomenon resonates with the common problem of not rejecting a null hypothesis and is not evidence of a true null hypothesis [74].

So far, we based our model on the assumption of multivariate normality (the presupposition that all the modelled variables have Gaussian marginal distributions and the relationships between them are linear). This can be relaxed by assuming that observed data come from a set of normally distributed latent variables. With this assumption in place, marginal distribution functions can be generated either by nonparanormal transformation [75] or threshold functions [76]. Nevertheless, assuming a Gaussian distribution of the latent variables, especially in clinical psychology, may still not be plausible in various cases. For instance, mental disorders, even in clinical samples, may not be normally distributed for several reasons. On the one hand, the prevalence of psychopathologies (e.g., suicidal behaviour or NSSI) may be lower than what a Normal distribution would predict. On the other hand, unlike in the Gaussian distribution, a value of zero is not just one from all possible values but means the absence of a psychopathological symptom. One potential solution for this is the dichotomisation of the problematic variables. If all variables are binary, an Ising Model can be estimated [77]. However, if the variables in the model are from different statistical distributions or families, Mixed Graphical Models (MGM; [78,79]) offer a solution. MGMs can also make use of the EBIC LASSO procedure.

MGMs allow each node to belong to different variable types; for instance, binary categorical and Gaussian. Earlier Gaussian-Ising models [80,81,82] were computationally expensive as they assumed that the distribution of the continuous variables conditioned on all possible configurations of the categorical ones followed a multivariate Gaussian distribution. Yang et al. (2014) proposed a model that combines distinct conditional distributions from the exponential family into one joint distribution. Accordingly, Haslbeck and Waldorp (2016) integrated this model into the covariance approach proposed by Loh and Wainwright (2013) [83].

In Hypothesis 3, we calculated the binary diagnosis variables both for the NSSI variable and for each mental disorder and, to reduce the number of variables due to the limited sample size, we combined them into six categories. As QoL dimensions are assumed to follow a Gaussian distribution and NSSI, as well as diagnoses of mental disorders being represented by binary categorical variables, we estimated MGMs including NSSI and the seven QoL categories as Gaussian variables and the NSSI variable, incorporating the six mental disorders as categorical binary variables. This model was implemented for R by the mgm package [78]; however, we carried out our analysis using the bootnet package [40], which is a wrapper for the mgm and the qgraph [84] packages. The latter contains the EBIC LASSO estimation. First, we estimated an MGM for only the NSSI and QoL dimensions; second, we added the mental disorders. We estimated the full model with a hyperparameter of 0.25 first, and then, as a robustness check, with six different hyperparameter values between 0 and 0.5.

## 3. Results

### 3.1. Sample

During the recruitment period, 257 patients (13–18 years) were treated in the Vadaskert Child and Adolescent Psychiatric Hospital and Outpatient Clinic. According to the exclusion criteria, 33 patients were not involved in our study. A further 22 refused to participate, thus the sample consisted of 202 participants. Data were missing for 11 participants (most of which comprised NSSI and QoL items), so the final study sample consisted of 191 clinical adolescents (girls: *n* = 95; 49.7%). The mean age was 14.85 years (SD = 1.39). In the study group, 98 adolescents (51.3%, girls: 68.4%) engaged in NSSI. 70.5% of girls and 32.3% of boys reported NSSI.

Table 1 shows the difference between NSSI and no-NSSI groups related to demographic variables. NSSI group contains adolescents engaged in NSSI, and no-NSSI group contains participants without NSSI. Except for gender, no significant difference was found between groups. Altogether 30.11% of the no-NSSI group and 68.4% of the NSSI group are female.

Following table (Table 2) shows the difference between NSSI and no-NSSI group related to mental disorders. There are significant more mood disorders, attention-disruptive disorders, substance use disorders, psychotic disorders and suicidality in NSSI group.

### 3.2. Self-Reported QoL Rating

There was a significant difference between the NSSI and no-NSSI groups related to family, physical health, mental health, and global well-being. Adolescents engaged in NSSI were rated significantly higher (meaning worse QoL) in these QoL areas (Table 3).

### 3.3. Self vs Parents’ Rating of QoL of Adolescents Engaged in NSSI

Parent and adolescent QoL domain scores were closer to each other in the NSSI sample than in the no-NSSI sample. There is no significant difference between self and parent QoL ratings among adolescents in the NSSI group. In the no-NSSI clinical group, parents rated their children as having a significantly higher QoL (meaning worse QoL) in the areas of peer relations, mental health, and global well-being (Table 4).

### 3.4. Associations between NSSI and QoL Dimensions

**Figure 1:**
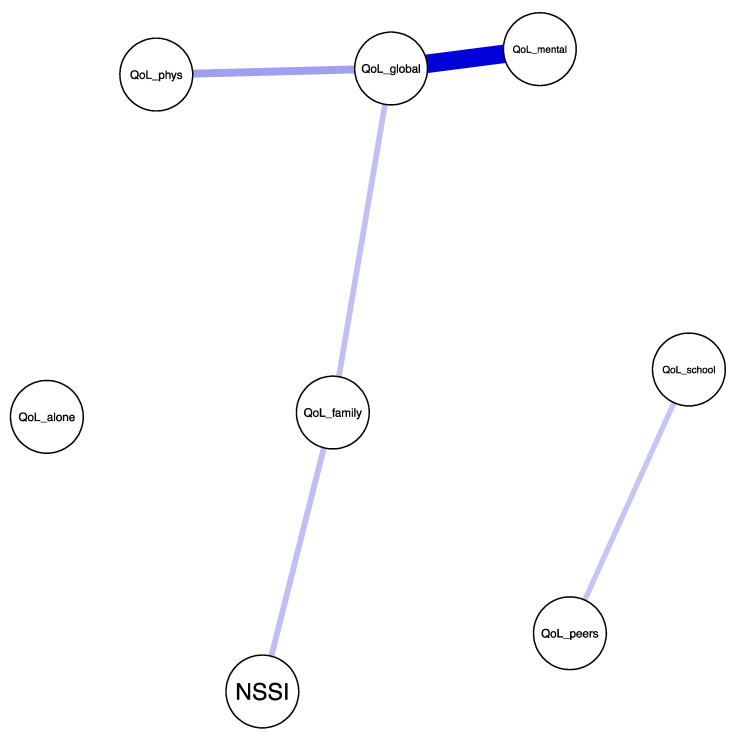
Association between QoL dimensions and NSSI. QoL_school—QoL school dimension, QoL_family—QoL family dimension, QoL_peers—QoL peer relations dimension, QoL_alone—QoL alone activity dimension, QoL_phys—QoL physical health dimension, QoL_mental—QoL mental health dimension, QoL_global—QoL global well-being dimension, NSSI—nonsuicidal self-injury.

The strongest positive correlation can be found between QoL mental health and QoL global well-being, *r* = 0.58, bootstrapped 95% CI [0.50, 0.67]. The second positive correlation is between the QoL family dimension and NSSI, *r* = 0.27, bootstrapped 95% CI [0.12, 0.46]. There is also a positive relationship between QoL physical health and QoL global well-being, *r* = 0.22, bootstrapped 95% CI [0.14, 0.32]. A positive association can also be found between the QoL school and QoL peer dimensions, *r* = 0.20, bootstrapped 95% CI [0.10, 0.32]. Furthermore, there is a positive relationship between the QoL family and QoL global well-being dimensions, *r* = 0.18, bootstrapped 95% CI [0.10, 0.28] (Figure 1).

### 3.5. Associations among NSSI, QoL, and Mental Disorders

**Figure 2:**
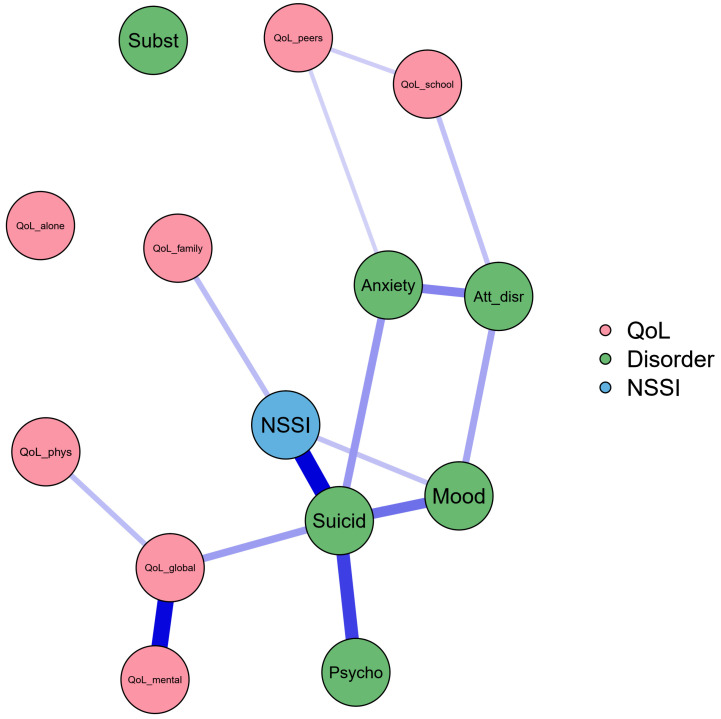
Association between QoL dimensions, mental disorders and NSSI. QoL_school—QoL school dimension, QoL_family—QoL family dimension, QoL_peers—QoL peer relations dimension, QoL_alone—QoL alone activity dimension, QoL_phys—QoL physical health dimension, QoL_mental—QoL mental health dimension, QoL_global—QoL global well-being dimension, NSSI—nonsuicidal self-injury, Mood—mood disorders, Anxiety—anxiety disorders, Att_disr—attention-disruptive disorders, Subst—substance use disorders, Psycho—psychotic disorders, Suicid—suicidality.

There is a positive significant relationship between NSSI, QoL dimensions, and mental disorders. The strongest correlation is between NSSI and suicidality, *r* = 0.61, bootstrapped 95% CI [0.22, 0.91]; the second is between QoL mental health and QoL global well-being, *r* = 0.57, bootstrapped 95% CI [0.46, 0.63]; the third closest relationship is between suicidality and psychotic disorders, *r* = 0.46, bootstrapped 95% CI [0.20, 0.10]; the fourth is between suicidality and mood disorders, *r* = 0.35, bootstrapped 95% CI [0.14, 0.69]. Respectively, the fifth to fifteenth most significant relationships were between attention-disruptive disorders and anxiety disorders, *r* = 0.30, bootstrapped 95% CI [0.18, 0.85]; the correlation between suicidality and anxiety disorders, *r* = 0.25, bootstrapped 95% CI [0.14, 0.67]; between QoL global well-being and suicidality, *r* = 0.24, bootstrapped 95% CI [0.15, 0.43]; between attention-disruptive disorders and mood disorders, *r* = 0.22, bootstrapped 95% CI [0.14, 0.61]; the correlation between QoL family and NSSI is *r* = 0.16, bootstrapped 95% CI [0.11, 0.41]; between QoL school and attention-disruptive disorders, *r* = 0.15, bootstrapped 95% CI [0.11, 0.42]; between NSSI and mood disorders, *r* = 0.15 bootstrapped 95% CI [0.11, 0.55]; between QoL school and QoL peers, *r* = 0.12, bootstrapped 95% CI [0.08, 0.29]; between QoL peers and anxiety *r* = 0.11, bootstrapped 95% CI [0.10, 0.42]; between QoL physical health and QoL global well-being *r* = 0.16, bootstrapped 95% CI [0.13, 0.29] (Figure 2).

**Figure 3:**
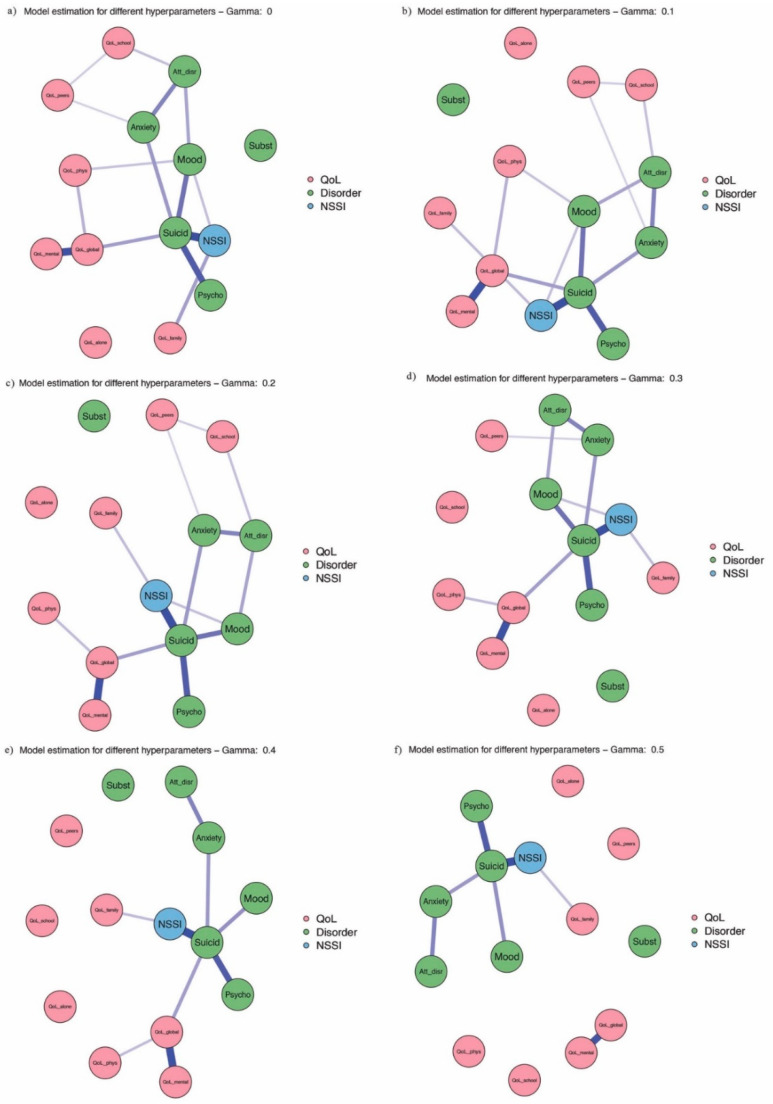
Robustness check-association between QoL dimensions, mental disorders and NSSI for different hyperparameters. QoL_school—QoL school dimension, QoL_family—QoL family dimension, QoL_peers—QoL peer relations dimension, QoL_alone—QoL alone activity dimension, QoL_phys—QoL physical health dimension, QoL_mental—QoL mental health dimension, QoL_global—QoL global well-being dimension, NSSI—nonsuicidal self-injury, Mood—mood disorders, Anxiety—anxiety disorders, Att_disr—attention-disruptive disorders, Subst—substance use disorders, Psycho—psychotic disorders, Suicid—suicidality.

Figure 3 investigates the robustness of the results representing the interrelationship between the variables using different hyperparameters. The more models contain a certain edge, the more robust the finding between two nodes. Globally, the composition of the networks does not change by altering the hyperparameter. First, the direct (weak) relationship between family problems and NSSI is consistent across all models. Second, psychosis–suicidality–NSSI seems to be a robust path as well. Both paths are present in all six networks. Third, both anxiety and mood disorders predict suicidality and, in turn, NSSI; attention-disruptive disorders predict anxiety disorders in all models, and they predict mood disorders in four of six. Additionally, mood disorders are a weak direct predictor of NSSI in four models. Fourth, peer and school problems are weakly correlated in three networks and the former is linked to anxiety four times, whilst the latter is linked to attention-disruptive disorders three times. Fifth, global QoL is strongly linked to the mental facet of QoL in all six models and is a weak-to-moderate predictor of suicidality and, in turn, of NSSI in five out of six networks. Furthermore, global QoL is a direct predictor of NSSI in one model. Sixth, the physical dimension of QoL is weakly linked to global QoL in five models and is also (weakly) linked to mood disorders in two networks. Finally, the alone activities dimension of QoL as well as substance abuse are consistently not associated with any of the other variables (after controlling for the effect of all other variables).

## 4. Discussion

To the best of our knowledge, this is the first study to investigate not only the agreement between the QoL ratings of self and parents in clinical adolescents who engaged in NSSI, but also the complex interrelations between QoL dimension, mental disorders, and NSSI. In doing so, it uses the framework of psychological networks that provides a more appropriate analytical toolkit than simple mediation analysis.

Our results are consistent with previous studies that have emphasised the high prevalence rate of NSSI within the adolescent population [7,8,9,10]. According to our results, more than half of this clinical sample of adolescents (51.3%) had engaged in some form of NSSI at some point in their life. Adolescents with NSSI reported higher rate of comorbid mental disorders like mood disorders, attention-disruptive disorders, substance use disorders, psychotic disorders and suicidality, than adolescents without NSSI. Our findings are in line with those of previous studies [12], which have suggested that NSSI is more common among women. In our study, 70.5% of girls and 32.3% of boys had engaged in NSSI.

In accordance with our first hypothesis, clinical adolescents who engaged in NSSI reported a lower QoL than adolescents without NSSI. Clinical adolescents who reported NSSI rated lower satisfaction in family life, physical and mental health, and global well-being than clinical adolescents without NSSI. This means that those adolescents in the clinical population who suffered from different symptoms of mental disorders rated their QoL significantly better than those clinical adolescents who also reported mental disorder symptoms and the aforementioned comorbid NSSI. According to our findings, adolescents who reported NSSI rated lower satisfaction in physical and mental health; this supports the notion that individuals who engaged in self-injury reported worse mental and physical health monthly than individuals who had not engaged in self-injury [34].

Lower family life satisfaction among adolescents with NSSI shows that family dysfunction might be especially important in this group. Our results, consistent with previous studies, emphasises that interpersonal problems predict the risk of NSSI [49,85]. Baetens et al. (2013) found that adolescents who engaged in NSSI reported more parenting behavioural and psychological control. Interestingly, their parents’ perception of their own parenting support, behavioural control, and psychological control did not differ significantly from other parents’ reports. Significant risk factors for NSSI are the high parental control and low support [86], poor general family functioning, low affective involvement [87], less protection, less care, less trust, less communication and more fear, more overprotection, and more alienation [88]. This family environment plays a vital role in the development of adaptive and maladaptive coping strategies [88]. Furthermore, previous studies also suggested that students who engaged in NSSI have a lower quality relationship with their parents [89], and a negative family relationship has an effect on emotional dysregulation, which can increase the occurrence of NSSI [90].

According to the opinion of young people, major protective factors which inhibit them from wanting to harm themselves are family, peer relations, and a supportive school environment. These are more likely to prevent such behaviour than external helping agencies [91]. Young people think that communication in the family is crucial, and parents should be educated related to self-injurious behaviour in order to have appropriate conversations regarding their children’s self-injury [91]. In addition, family also plays an important role in supporting and accepting professional treatments for youth engaged in NSSI [92]. Effective interventions for NSSI treatment consider family dynamics, related contextual factors, supporting familial and other interpersonal relationships, the improving of parenting skills, and the development of adolescents’ coping skills [93].

An interesting finding from our study related to our second hypothesis is that there is no significant difference between self and parent QoL evaluations in the NSSI sample, while in the no-NSSI sample parents rated peer relations, mental health, and global well-being for their children to be significantly worse than their children did. The parents of those adolescents who engaged in NSSI rated better physical health but worse global well-being for their children compared to the adolescents’ evaluations. Nevertheless, the differences in the evaluations are not significant.

Higher parental ratings of the negative effects of mental disorders have been demonstrated in several studies. In a previous study, mothers of depressed children rated the QoL of their children to be significantly lower than the children themselves [26]. Bastiaansen et al. (2004) investigated children with psychiatric disorders and found that parents rated their children’s QoL to be lower in all measured psychiatric disorders and also in the case of children without any psychiatric diagnosis [21]. Danckaerts et al. (2010) suggested that children with ADHD rated their QoL less negatively than their parents did [24]. In case of the no-NSSI sample, our findings are in line with a previous study showing that the agreement on QoL ratings between children and their parents was moderate [21]. In contrast to the aforementioned literature results, we found no significant difference between self and parent QoL evaluations in the NSSI sample.

According to our results, the agreement between self and parents was better in the sample of adolescents who engaged in NSSI as compared to the no-NSSI sample. Our results in the no-NSSI sample identifies that when children had a somatic or mental disorder, parents rate the effects of the illness more negatively than the children themselves [26]. This exception to this is when NSSI also occurs; in our NSSI sample, this effect does not exist, as the adolescents’ self-evaluation and their parents evaluation of QoL are similar. Symptoms of mental disorders are often less observable than the symptoms of physical illnesses, and this might explain why the self and parent rating agreements is worse in the case of psychiatric illnesses [26]. NSSI is a direct, deliberate destruction of one’s own body tissue without suicidal intent [94], so it could be observable on the body. NSSI impacts the entire family life, and this effect could be bidirectional [95,96]. NSSI may also influence parental wellbeing, their parenting, and their daily life and behaviour related to providing support for their children’s needs [95,97]. Several studies have confirmed in community samples that parents do not have clear images related to their child’s NSSI behaviour [95,96], and they also underestimate the frequency and the onset of NSSI [96]. In community samples, approximately one in three caregivers know that their adolescent child engaged in NSSI [95].

As only a clinical sample of adolescents took part in our study, we suppose that parents had direct information about the exact psychiatric status of their child. In general, when parents recognise that their child engaged in NSSI, they often feel sadness, anger, guilt, confusion, and shock [87,97,98,99]. Parents often think that their child tried to commit suicide, and NSSI is a form of suicide attempt [92]. After the first shock, parents often tend to use more positive parenting behaviour, and more support and monitoring. These positive parenting behaviours could, however, also have a significant effect on NSSI. In addition, the presence of NSSI might also increase parental controlling behaviours and punishment [95]. Teenage years can be challenging both to adolescents and their parents. In this time, adolescents need both autonomy and connectedness, as well as familial attachment relationships [20]. According to the NSSI Family Distress Cascade Theory [20], when NSSI is revealed, parents often apply more control over adolescents, but this decreases their sense of autonomy and connectedness, and in order to gain the sense of autonomy again, adolescents increase the frequency of NSSI. In turn, this results in more and more parental controlling behaviour because parents fear suicide and they want to know that their child is fine; however, the whole family feels distressed [20]. This theory emphasises that there is a bidirectional, dynamic relationship between the adolescents who engage in NSSI and their caregivers’ reactions [20]. This increased parental controlling behaviour might contribute to our results that in the NSSI group no significant difference was shown between self and parent reports on QoL ratings.

Other alternative explanation could be the communication function of NSSI. Problems with affect regulation and interpersonal communication mean risk for NSSI. Without adaptive social-problem solving and communication skills NSSI might be a seemingly effective communication form in an unresponsive environment. Adolescents who engage in NSSI may be incapable to signal their feelings and need for help appropriately to their social environment [100].

As for the third hypothesis, our findings are mixed so we cannot arrive at a clear-cut conclusion regarding the mediating effects. We hypothesised that the association between mental disorders and NSSI is mediated by QoL. However, the network model showed that this hypothesis is not supported; most of the QoL dimension (except for family problems) are conditionally independent from NSSI if one controls for mental disorders. In other words, the relationship between NSSI and QoL is mediated by mental disorders.

Previous studies have emphasised the association between dissatisfaction with life and DSH [32,34,35]. In our model, in all QoL dimensions only the family area has a direct relationship with NSSI, which emphasises the relevant role played by family life concerning NSSI occurrence [20,95,96].

From all QoL areas and mental disorders above the QoL family dimension, suicidal behaviour and mood disorders have a direct association with NSSI occurrence. All other QoL domains and mental disorders have a relationship with NSSI only through suicidal behaviour; this is the strongest association found between NSSI and suicidal behaviour. NSSI is, therefore, a risk factor for suicidal behaviour [16]. Suicide is also a fundamental concern for youth (15–29 years of age), because it is the second most common cause of death after traffic accidents in this age group [101]. These two phenomena are strongly associated and the comorbidity prevalence rate in adolescent psychiatric samples is approximately 60–80% [9,10]. Adolescents with NSSI and suicidal behaviour are diagnosed more frequently with mental disorders, and family life problems are more common among these young people than problems in their peer relationships [102].

In our results, there is a link between QoL evaluation of peer relationships and anxiety disorders. A systematic review and meta-analysis in adolescents reveal that friendship quality, peer rejection, and victimisation is associated with later social anxiety, and there is a bidirectional relationship between peer functioning and social anxiety [103]. Peer relation is associated indirectly with NSSI through anxiety disorders and suicide. This means that the lower QoL of peer relationships has a direct association with anxiety disorders, which also have a direct link to suicidal behaviour; through suicidal behaviour, this is connected to NSSI. Vergara et al. (2019) found that those adolescents who engaged in NSSI, and have current suicide ideation and one or more lifetime suicide attempts, reported higher levels of peer victimisation [104]. According to our findings, among mental disorders only suicidal behaviour and mood disorders have a direct positive association with NSSI, and other mental disorders like anxiety disorders and psychotic disorders have an indirect relationship with NSSI through suicidal behaviour. The relationship between NSSI and mood disorders is much weaker than the association between NSSI and suicidal behaviour. Anxiety disorders are one of the most common mental disorders in children and adolescents [105] and are highly comorbid with other psychiatric symptoms like ADHD, ODD, dysthymic disorders, and major depressive disorders [106]. Our results supported that there is a relationship between anxiety disorders and suicide risk [107]. According to our findings, a stronger link can be found between mood disorders and suicide than between anxiety disorders and suicidal behaviour, which supports that one of the strongest predictors for suicidal behaviour is major depressive disorder [108].

In our model, we can see a longer pathway between QoL school life area and NSSI. The QoL evaluation of school life has a direct relationship with attention-disruptive disorders, which has a direct association with mood disorders; this has a direct link to suicidal behaviour, which has a direct relationship with NSSI. Accordingly, the lower QoL of school life associated with attention disruptive disorders, which has a comorbidity with mood disorders, can lead to suicide and NSSI. With the exception of substance use, our finding is consistent with those previous findings suggesting that clinical adolescents with ADHD have a high risk of suicidal behaviour, and this relationship is fully mediated by depression, dysthymia, and substance use [109]. In our study, attention-disruptive disorders have no direct link to NSSI, which is in line with previous findings reporting that there is no direct relationship between the symptoms of ADHD and NSSI; however, this relationship is fully mediated by comorbid mental disorders [48]. Our results are similar to those of a previous study from our group in which we found that QoL did not mediate the relationships between hyperactivity/impulsivity/conduct symptoms and suicidal risk; furthermore, hyperactivity/impulsivity symptoms had a relationship with more emotional symptoms and conduct problems, and more emotional symptoms led to a higher level of suicidal risk [31].

According to our findings, in clinical populations, adolescents’ global well-being evaluation is directly based on how they feel physically and mentally. Whereas the association between physical health and global well-being is relatively weak, the mental health-global well-being relationship is so strong that discriminant validity between the two constructs might be questionable. In a previous study, it was also found that sleep quality influences physical and mental well-being, which predicted global QoL [110]. Consequently, the QoL evaluation of physical health and mental health influences how they feel in general (QoL global well-being area) and this has an indirect association with NSSI through suicidal behaviour. In our study, there is a direct positive relationship between global well-being and suicidal behaviour. This is consistent with previous findings which reported lower QoL for a psychiatric population of adult patients with suicidal risk and revealed that mood disorders, psychotic syndromes, and anxiety disorders are especially common comorbidities in this group [111].

To wrap up our main findings, with the exception of the family related QoL dimension, QoL is not directly related to NSSI. Hence, as opposed to our third hypothesis, according to which QoL mediated the relationship between mental disorders and NSSI, our results show that actually mental disorders serve as mediators in the QoL-NSSI relationship. Assuming that NSSI is an outcome in our models, the two results are qualitatively different. Whereas in the original hypothesis (genetic) predispositions (mental disorders) lead to QoL impairments that, in turn, result in NSSI, our results show that environmental factors (QoL problems) cause mental disorders that, in turn, bring about self-injury. Although we did not test it directly, both the disorder-QoL-NSSI and the QoL-disorder-NSSI models would likely have yielded significant mediation effects. We could rule out the first option because we estimated a model that did not pre-specify whether a variable (node) is an IV, mediator, or DV. This result warns against the use of classical mediation analysis that is supposed to reveal the mechanisms behind certain relationships. The use of classical mediation analysis has been criticised even in the analysis of experimental data [112] because if a variable is affected by the IV and can be confounded (shares method variance) with the DV, it is a perfect candidate for being a mediator even if it does not explain the actual mechanism. This problem can become particularly serious when analysing non-experimental data where one does not know anything about causality. Hence, network modelling may provide a better framework for explaining the mechanisms behind multi-dimensional data.

Our results need to be interpreted with the consideration of several limitations. First, due to the cross-sectional study design, although we could estimate conditional independence relationships, we do not know the exact direction of causality. NSSI was treated as an outcome measure and we aimed to explore its predictors, as well as the relationship between them. Although it is not a particularly plausible mechanism, it is possible that NSSI leads to mental disorders that, in turn, impair QoL. Alternatively, the model might signal that both NSSI and QoL impairment cause mental disorders. A somewhat more likely mechanism is that mental disorders lead to either NSSI or QoL impairment. Nevertheless, among all possible causal pathways, we consider our explanatory mechanism to be the most persuasive. In future studies, alternative explanations should be investigated by the use of time-series analysis. Second, we used self-rating scales for the assessment of QoL and NSSI. Third, because of the small sample size, our results must be considered as a preliminary study. Future research should explore the possible way between NSSI, QoL, and mental disorders with network approach in larger clinical and non-clinical samples. Longitudinal studies are required to clarify the causal relationship. Moreover, mental retardation was an exclusion criterion in our study, but we did not use any IQ measure, and our information was based only on patients’ history. Finally, we did not use any investigation related to parents’ actual psychopathology and well-being, which can influence parents’ opinions about their child’s QoL [113,114].

## 5. Conclusions

In summary, our study supports those previous studies which have suggested high NSSI prevalence in clinical populations of adolescents. Adolescents who reported NSSI also disclosed lower QoL than adolescents without a history of NSSI. Our results highlight that mental disorders mediate the relationship between lower QoL and NSSI occurrence. According to our results, agreement between self and parent QoL ratings was similar in adolescents who engaged in NSSI. Early recognition of lower QoL can contribute to the prevention of mental disorders which can lead to higher NSSI occurrence. Based on our data, NSSI prevention strategies should involve the routine assessment of QoL in adolescents, especially in clinical settings. QoL could, therefore, be an important outcome variable to assess the efficiency of NSSI treatment [115].

## Figures and Tables

**Table 1:** Difference of demographic variables between NSSI and no-NSSI group.

Demographic Variables	Chi/*t*-Value	*p*-Value	Corrected *p*-Value (Bonferroni)	*df*
Father’s education level	0.770	0.680	1.000	2.000
Mother’s education level	1.741	0.419	1.000	2.000
Father’s job market status	7.572	0.144	1.000	5.000
Mother’s job market status	4.932	0.584	1.000	6.000
Family’s economic situation	0.534	0.581	1.000	1.000
Family type	3.665	0.641	1.000	5.000
Adoption status	2.469	0.757	1.000	4.000
Mental problem in family	8.912	0.059	1.000	4.000
Suicide in family	8.043	0.097	1.000	4.000
Number of siblings	4.686	0.802	1.000	8.000
Number of stepsiblings	0.553	0.512	1.000	1.000
Twin	1.205	0.818	1.000	2.000
Pregnancy	0.000	1.000	1.000	1.000
Birth details (complication)	0.070	0.870	1.000	1.000
Birth time (too early/late)	2.799	0.280	1.000	2.000
Early childhood (problems)	0.196	0.816	1.000	1.000
Left/right-handed	1.829	0.405	1.000	2.000
Psychological treatment ever	0.830	0.433	1.000	1.000
Chronic illness	1.454	0.320	1.000	1.000
Child is taking medication	0.017	1.000	1.000	1.000
Child was taking medication	0.197	0.719	1.000	1.000
School (or workplace) type	3.495	0.528	1.000	4.000
Age (Welch two-sample *t*-test)	0.065	0.948	1.000	188.460
Sex	27.940	0.000	0.012 *	1.000

NSSI group—adolescents engaged in NSSI, no-NSSI group—adolescents without NSSI, Chi/*t*-values: Chi-square test, T-test, *p*-Value—*p*-value of significance, Corrected *p*-Value—*p*-value of significance with Bonferroni correction, *df*—degrees of freedom, * *p* < 0.05.

**Table 2:** Mental disorders in NSSI and no-NSSI group.

NSSI–Mental Disorders	NSSI		No-NSSI		Chi-Squared Statistics	*p*-Value	Corrected *p*-Value (Bonferroni)
	Mean	SD	Mean	SD			
Mood disorders	0.602	0.492	0.290	0.456	38.441	0.000	0.000 ***
Anxiety disorders	0.796	0.405	0.591	0.494	3.660	0.056	0.334
Att. disr. disorders	0.480	0.502	0.280	0.451	12.392	0.000	0.002 **
Substance use disorders	0.235	0.426	0.118	0.325	8.497	0.004	0.022 *
Psychotic disorders	0.265	0.444	0.065	0.247	7.260	0.007	0.042 *
Suicidality	0.684	0.467	0.226	0.420	17.494	0.000	0.000 ***

NSSI group—adolescents engaged in NSSI, no-NSSI group—adolescents without NSSI, Mean—mean value, SD—standard deviation, Chi-Squared Statistics—Chi-square test, *p*-Value—*p*-value of significance, Corrected *p*-Value—*p*-value of significance with Bonferroni correction, * *p* < 0.05, ** *p* < 0.01, *** *p* < 0.001.

**Table 3:** Adolescents’ self-reported QoL rating.

Domains of Quality of Life (QoL)	Mean (NSSI)	Mean (No-NSSI)	*t*-Value	*df*	*p*-Value	Corrected *p*-Value (Bonferroni)
School	3.286	2.957	−1.895	184.315	0.060	0.418
Family	2.704	2.032	−4.517	186.371	0.000	0.000 ***
Peer relations	2.429	2.172	−1.517	188.569	0.131	0.917
Alone activity	2.143	1.796	−2.310	188.017	0.022	0.154
Physical health	2.714	2.194	−3.400	188.809	0.001	0.006 **
Mental health	3.796	3.151	−3.972	188.063	0.000	0.001 **
Global well-being	3.378	2.667	−4.508	188.203	0.000	0.000 ***

NSSI group—adolescents engaged in NSSI, no-NSSI group—adolescents without NSSI, Mean—mean value of QoL score. Lower ratings represent better quality of life (1: very good, 2: fair enough, 3: medium, 4: rather bad, 5: very bad). *t*-Value—T-tests, *df*—degrees of freedom, *p*-Value—*p*-value of significance, Corrected *p*-Value—*p*-value of significance with Bonferroni correction, ** *p* < 0.01, *** *p* < 0.001.

**Table 4:** Adolescents’ self- and parent reported QoL rating.

Domains of Quality of Life	NSSI						No-NSSI					
	Mean (Self-Rating)	Mean (Parent Rating)	*t*-Value	*df*	*p*-Value	Corrected *p*-Value (Bonferroni)	Mean (Self- Rating)	Mean (Parent Rating)	*t*-Value	*df*	*p*-Value	Corrected *p*-Value (Bonferroni)
School	3.286	3.337	−0.416	97.000	0.678	1.000	2.957	3.226	−2.056	92.000	0.043	0.298
Family	2.704	2.602	0.799	97.000	0.426	1.000	2.032	2.215	−1.727	92.000	0.088	0.613
Peer relations	2.429	2.622	−1.271	97.000	0.207	1.000	2.172	2.527	−3.169	92.000	0.002	0.015 *
Alone activity	2.143	2.071	0.487	97.000	0.627	1.000	1.796	2.043	−1.885	92.000	0.063	0.438
Physical health	2.714	2.378	2.737	97.000	0.007	0.052	2.194	2.172	0.188	92.000	0.851	1.000
Mental health	3.796	3.949	−1.315	97.000	0.191	1.000	3.151	3.688	−4.247	92.000	0.000	0.000 ***
Global well-being	3.378	3.673	−2.608	97.000	0.011	0.074	2.667	3.419	−6.850	92.000	0.000	0.000 ***

NSSI group—adolescents engaged in NSSI, no-NSSI group—adolescents without NSSI, Mean—mean value of QoL score. Lower ratings represent better quality of life (1: very good, 2: fair enough, 3: medium, 4: rather bad, 5: very bad). *t*-Value—T-tests, *df*—degrees of freedom, *p*-Value—*p*-value of significance, Corrected *p*-Value—*p*-value of significance with Bonferroni correction, * *p* < 0.05, *** *p* < 0.001.

## Data Availability

The data presented in this study are available on request from the corresponding author, without undue reservation, to any qualified researcher.
